# A System to Support Diverse Social Program Management

**DOI:** 10.2196/23219

**Published:** 2021-08-30

**Authors:** Mollie McKillop, Jane Snowdon, Van C Willis, Shira Alevy, Rubina Rizvi, Karen Rewalt, Charlyne Lefebvre-Paillé, William Kassler, Gretchen Purcell Jackson

**Affiliations:** 1 IBM Watson Health Cambridge, MA United States; 2 Vanderbilt University Medical Center Nashville, TN United States

**Keywords:** other clinical informatics applications, process management tools, requirements analysis and design, consumer health informatics, public health

## Abstract

**Background:**

Social programs are services provided by governments, nonprofits, and other organizations to help improve the health and well-being of individuals, families, and communities. Social programs aim to deliver services effectively and efficiently, but they are challenged by information silos, limited resources, and the need to deliver frequently changing mandated benefits.

**Objective:**

We aim to explore how an information system designed for social programs helps deliver services effectively and efficiently across diverse programs.

**Methods:**

This viewpoint describes the configurable and modular architecture of Social Program Management (SPM), a system to support efficient and effective delivery of services through a wide range of social programs and lessons learned from implementing SPM across diverse settings. We explored usage data to inform the engagement and impact of SPM on the efficient and effective delivery of services.

**Results:**

The features and functionalities of SPM seem to support the goals of social programs. We found that SPM provides fundamental management processes and configurable program-specific components to support social program administration; has been used by more than 280,000 caseworkers serving more than 30 million people in 13 countries; contains features designed to meet specific user requirements; supports secure information sharing and collaboration through data standardization and aggregation; and offers configurability and flexibility, which are important for digital transformation and organizational change.

**Conclusions:**

SPM is a user-centered, configurable, and flexible system for managing social program workflows.

## Introduction

Government and community-based organizations are responsible for delivering social services to clients through social programs provided at the national, state, county, and city levels. Social programs are critical to the health and welfare of many citizens, as they provide a wide range of benefits [1], such as (1) health and human services for health insurance, prevention services, child welfare, and nutrition assistance; (2) workforce services for unemployment insurance programs and job training; and (3) social security programs offering income support and benefits [2].

In contrast to commercial entities, government agencies administering social programs face unique challenges regarding service delivery and their operational processes, including (1) information silos that limit decision-making abilities, (2) requirements to balance privacy with data sharing and transparent use of public funds, (3) reduced financial resources but growing demand for services, (4) legislative and organizational influences on eligibility and entitlement, and (5) the need to document the delivery of mandated services for beneficiaries across multiple categorical programs [3,4]. These challenges are exacerbated by societal shifts, such as increasing income inequality, aging populations, unemployment, ongoing changes in government policies, and resource constraints [5-7].

Opportunities exist for information technology to address these challenges by improving the efficiency and transparency of social program workflows and enabling collaboration among stakeholders. These goals can be achieved by centralizing critical data, streamlining eligibility determination and case management, and improving communication within and across organizations [8]. Previous preliminary research indicates that the use of information systems to support high-quality and efficient service delivery is promising but limited in scope and does not meet the unique needs of social programs [8-10]. Comprehensive systems specifically designed for social programs have not been previously described in the literature.

This viewpoint describes the design, functionality, and selected applications of a software solution for social services, which combines domain-specific business processes with a flexible open architecture to allow for needed configurability while standardizing data elements. Specific design principles that aim to address the unique challenges encountered by social programs, along with examples of implementation and usage, are described.

Our system was designed and developed by subject matter experts in social programs, including industry specialists, service professionals experienced in social programs, and social program product developers. The system was developed for social service and human service agencies to advance digital transformation, intending to support a wide range of constituents across the health and human services enterprise. Potential users include, but are not limited to, Medicaid program managers, directors, analysts, caseworkers and care managers, clients, and beneficiaries. The system aims to prioritize the needs of users and beneficiaries to unify multidisciplinary teams. User-centered research methods, including user shadowing, interviews, surveys, and scenario testing, were used to identify user needs, and human-centered design leveraging iterative co-development and prototyping were used to develop the system.

## System Scope

**Figure 1:**
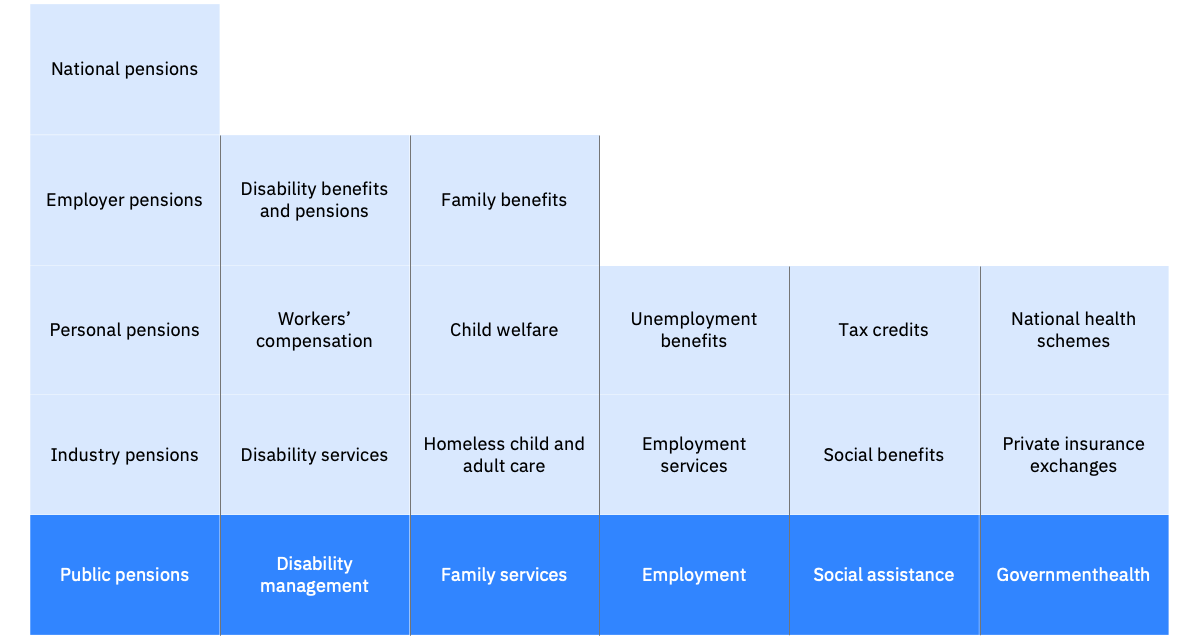
Scope of services supported by Social Program Management. Social Program Management serves health and social service organizations at all levels of government and nonprofit organizations.

Social Program Management (SPM) supports two basic types of social programs: (1) programs in which eligibility is determined primarily based on need and (2) programs where eligibility for benefits and services is determined based on previous contributions [11,12]. SPM also supports care and protection programs, such as child welfare programs, where eligibility is based on practice models and assessments; Figure 1 describes the full scope and scale of service organizations that SPM supports.

These diverse social programs share similar goals and challenges with the aim of improving service delivery to and outcomes for beneficiaries. To meet these goals, social programs require systems that (1) support complex eligibility and entitlement, (2) provide beneficiaries with easy access to services, (3) enable efficient management of high case volumes, (4) provide decision support and knowledge management tools, (5) allow flexibility for changes in policies and processes, and (6) reduce the potential for fraud and abuse. These needs must be met throughout triage, initial contract and registration, determination of eligibility for benefits, service planning and delivery, and outcome evaluation. Delivering services across these program stages relies on secure data gathering, documentation, retrieval, validation, auditing, and analysis. SPM has features and capabilities that address each of these needs (Table 1) [13].

**Table 1:** Features and capabilities to address needs.

Challenge and need			Features and functionalities
**Information and programs are managed in silos**			
	Integrate service delivery	Centralized repository for the integrated management of cases maintained in the system and external systems Multidisciplinary team portal provides a means for cross-agency and cross-program teams to collaborate on cases Indexing on cases and participants provides master data management, which allows for identifying individuals across systems, especially web services Citizen portal and multichannel access allows clients to check on status, submit applications, and manage benefits on agency websites	
	Consolidate infrastructure	The system data model supports needs-based and contribution-based programs Program-specific modules for social security, health and human services, and workforce services based on a common data model	
	Provide standards-based integration	The system data model can be deployed as web services Preconfigured adapters and enterprise application integration connectors to facilitate integration with existing systems	
**Data protection and privacy**			
	Prevent unauthorized access to sensitive data	Role-based and data field level security for personal and case data	
	Protect the integrity and privacy of personal data while sharing data responsibly	Configurable security levels to prevent unauthorized access to data while allowing multiple stakeholders to view a client’s data with the appropriate level of access Auditing and tracking of transactions involving sensitive data Auditing and traceability for logging time, date, and the user responsible for any read, update, and delete actions for any specified participant or case data elements	
	Reduce inaccurate or duplicate data	Integrated case management module supports centralized or distributed maintenance and sharing of case and participant data across programs	
**Shrinking budgets**			
	Provide standard programs and eligibility and entitlement rules with opportunities for customization	Eligibility and entitlement rules for income support and assessments for child welfare programs Configurable, packaged connectors and adapters for integration to existing and service-oriented applications	
	Leverage and consolidate cost-effective, existing infrastructure to deploy new program solutions	Support for open standards and de facto standards to ensure deployment on the widest possible range of operating systems, hardware platforms, and middleware	
	Provide tools to allow incremental approaches to implementation	Web services and configurable business processes allow for maximum flexibility in deployment and implementation options	
**Increasing demand for services**			
	Deliver high performance and scalability	Eligibility and entitlement engine optimized for processing and calculating high volumes of rules-based assessments and complex reassessments where information may need to be retroactively changed for large populations of clients Supervisor workspace for real-time analysis and dynamic allocation of workloads across a department or agency	
	Offer broad access and reliability	Multichannel access through a device-independent web-based user interface Configurable citizen portal for access to cross-program screening and eligibility	
**Legislative and organizational change**			
	Supply configurable systems that can adapt to legislation without reprogramming and support different organizational structures	Configurable eligibility and entitlement engine supporting complex reassessments Configurable regional administration segregates duties by location or organization Concurrent execution of reassessment batch jobs with ongoing web-based transactions	
**Balancing accuracy, consistency, and outcome focus**			
	Comply with legislation in the delivery of benefits across large populations while also individualizing services for clients and families	Integrated service-planning templates based on best practices, such as structured decision-making assessments, which are developed by the National Council on Crime and Delinquency Intelligent evidence gathering module scripts and templates for consistent, structured capture of information for caseworkers and beneficiaries Decision assist module, a configurable rules-based matrix designed to assure consistency and accuracy in rendering decisions The social enterprise collaboration module provides a common platform and set of tools for multidisciplinary collaboration in social program organizations	

## System Architecture

### Overview

**Figure 2:**
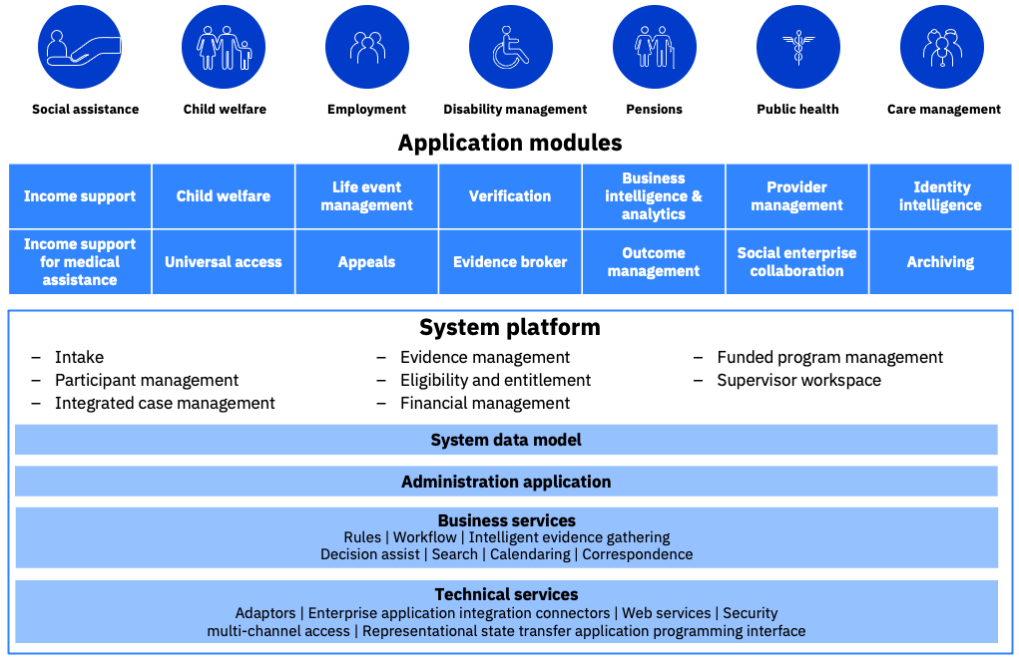
Social Program Management design. Social Program Management provides core processing and infrastructure components for social program management through its platform. Business services provide support for management needs common across all types of organizations. Modules complement the system platform and provide additional functionality and system configurability.

The features and functionalities described in Table 1 are organized within a single user-centered system comprising modules, the SPM data model, administration application, and business and technical services. The architecture for SPM version 7.0.9, the latest version, is outlined in Figure 2, and the modules and applications of the SPM are described in the following sections (see Multimedia Appendix 1 for technical aspects of SPM).

### SPM Modules

The SPM modules presented in Table 2 support repeatable processes that are common across programs. These processes include managing client information and data regarding benefits, automatic assessment of eligibility and entitlement, management of tasks, communication, and scheduling. Each module is supported by the SPM platform, including the data model and administrative, business, and technical services. The SPM modularization supports the incremental modernization of systems used by social programs. As the data model is application-agnostic, the integration of future modules or functionalities is supported. These modules support a comprehensive range of functionalities required for service delivery in social programs. Modules are divided by those that exist for all implementations of SPM (enterprise-wide applications) and those specific to a particular implementation and user agreement (implementation-specific modules).

**Table 2:** Social Program Management modules and applications.

Name	Type	Description
Intake	Enterprise-wide	The intake module facilitates integration with external systems that manage the client. The intake module notifies an external system when an intake is approved. The external system can then retrieve the details of the intake for further processing.
Participant management	Enterprise-wide	A participant is the system term for any individual or organization about which a social enterprise wants to record information. Participant management provides for the creation and maintenance of all relevant basic information such as contact details, addresses, communications, demographic information, and alternate names and identifiers.
Integrated case management	Enterprise-wide	Integrated case management provides functionality to facilitate the creation, management, and tracking of cases in support of social program service delivery. Interactions between participants and the agency and any associated program or service delivery are recorded. These interactions include assessment, eligibility determination, case approval, program delivery, outcome evaluation, and closure.
Evidence management	Enterprise-wide	Evidence is any data collected in support of a case. In general, such information is program-specific, although various types of evidence may be shared through a number of programs. Typically, the primary use of evidence is for the determination of program eligibility and entitlement. Evidence management provides capabilities to standardize and simplify the process of defining, creating, and maintaining such program-specific, temporal data.
Eligibility and entitlement	Enterprise-wide	The rules for determining both eligibility and entitlement are typically dictated by a combination of legislation, policy, and operating procedure. For many programs, such rules are determined by federal, state, or local governments. These rules often change. The system provides two mechanisms to help social enterprises deal with the problem of changing evidence: The ability to detect the change and the ability to initiate a reassessment where required Overpayment and underpayment processing where the system compares the old and new situations automatically detects any overpayments or underpayments and initiates appropriate action
Financial management	Enterprise-wide	Financial management manages and tracks the financial transactions associated with program delivery, including benefit payments and liability recovery. Financial management generates, manages, and tracks the financial transactions associated with cases and participants. It also supports the issue of payments and the creation of liabilities as determined by assessment and entitlement processing.
Funded program management	Enterprise-wide	Funded program management is used to manage funds that can be obligated to clients in need of assistance or to provide payment to providers for services.
Supervisor workspace	Enterprise-wide	Supervisor workspace provides dashboard-style views of a social program’s workload. It allows managers to monitor workloads through supervisor dashboard views of staff assignments, real-time display and access to information, centralized management of cases and tasks, prioritization, and allocation of workloads.
Income support	Implementation-specific modules	Income support delivers health and social program components, business processes, toolsets, and interfaces on a dynamically configurable architecture that allows an administrator to change rules without writing code. Income support is designed for programs that provide food, cash, and medical assistance.
Child welfare	Implementation-specific modules	Child welfare provides case management tools that support agencies that work to safeguard children, promote well-being, and support child permanency. Child services facilitate intake, ongoing case management, child abuse investigations, removal of children from unsafe situations, and the adoption of children.
Life event management	Implementation-specific modules	Life event management helps the caseworker to collect evidence and provide guidance that is based on a client’s life event, such as the birth of a child, marriage, divorce, or change in employment.
Verification	Implementation-specific modules	The verification engine streamlines the process of verifying evidence that is used in determining eligibility and entitlement as part of program delivery. It provides the functions that are needed for efficient management of verifications where policy or legislation mandates that evidence is verified as a prerequisite for eligibility.
Business intelligence and analytics	Implementation-specific modules	Business intelligence and analytics is a decision support solution that helps social program organizations analyze the effectiveness of their programs and gain insight into the efficiency of their operations. It is scalable from the program to enterprise level. It consists of embedded analytics, domain-specific dashboards, extract, transform, and load functions, and tool-independent, predefined, domain-specific (only for social program management) data marts.
Provider management	Implementation-specific modules	Provider management manages the interactions between the agency and its outside providers, such as foster families, housing facilities, and other vendors.
Identity intelligence	Implementation-specific modules	Identity intelligence aims to give caseworkers the confidence that an applicant is who they say they are and are not duplicated within the system, which might result in duplicate benefits. The product solves this problem by applying analytics to client data and verifies information with limited worker involvement.
Income support for medical assistance	Implementation-specific modules	Income support for medical assistance is specifically built to provide business tools and processes for the management of traditional medical assistance programs, plus the Affordable Care Act and modified adjusted gross income-based Medicaid programs.
Universal access	Implementation-specific modules	Universal access is a fully configurable web-based citizen-facing application that enables agencies to offer a web self-service solution to their clients. Universal access can provide a greater number of clients with access to programs and services by allowing clients to complete key tasks on the web without the assistance of a worker.
Appeals	Implementation-specific modules	Appeals is an automated solution that provides support for the appeals and fair hearings process. Appeals automates the intake, hearings, and decision processes and manages participants in the appeals process. Appeals supports multilevel appeals in which multiple issues for an appellant and respondent can be viewed at a single appeal hearing.
Evidence broker	Implementation-specific modules	Evidence broker facilitates flexible data sharing between different case types and between agencies.
Outcome management	Implementation-specific modules	Outcome management provides organizations that deliver social programs with a framework and automated tools to create and manage outcome plans for clients and their families. Outcome management is designed to help organizations assess needs, establish goals, plan for goal attainment, and track progress.
Social enterprise collaboration	Implementation-specific modules	Social enterprise collaboration is a common platform and set of tools for multidisciplinary collaboration in social programs. Multidisciplinary teams are involved in supporting the needs of clients and families, including other agencies, local providers, and interested community partners.
Archiving	Implementation-specific modules	As database size grows, performance can degrade rapidly. A large percentage of data in a social program database is unlikely to be accessed daily. Performance can be greatly improved if infrequently accessed data are removed from the production environment. Archiving stores and maintains inactive data in a repository so that it can be retrieved when necessary.

## System Usage and Case Reports

**Figure 3:**
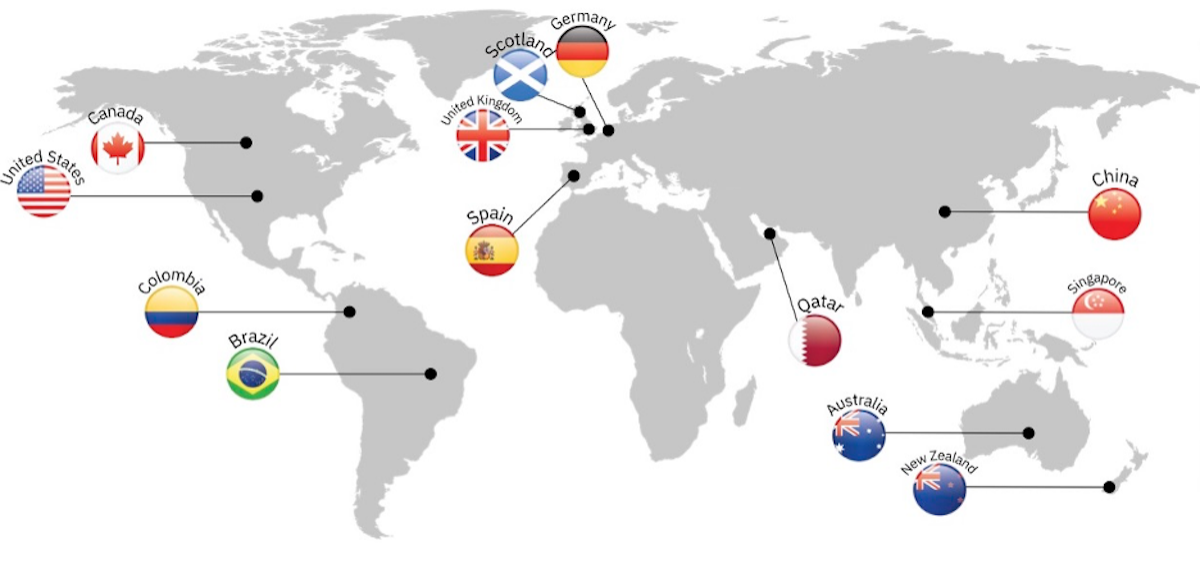
Worldwide usage of Social Program Management.

The latest version of SPM has been used by more than 50 programs in 13 countries (Figure 3) and 13 languages. In total, more than 280,000 caseworkers worldwide have used SPM with 30 million beneficiaries.

### Case Report 1: User-Centered Design for Social Program Efficiency

Eligibility and entitlement determination are often complex, time-consuming, and one of the most frustrating aspects of social program service delivery for administrators and clients. A large US city department of human resources used SPM’s universal access module to redesign their Supplemental Nutrition Assistance Program (SNAP) [14] to digitize services and reduce wait times compared with manual processing of applications and limit the need for in-person visits.

In April 2018, the city implemented a citizen-facing portal, available in 7 languages, to deliver the SNAP and other benefits via clients’ own desktop and mobile phones. The design of the portal was informed by 30 shadowing sessions, 10 interviews, and 20 scenario testing sessions. Iterative prototyping and co-design workshops were also conducted to refine the portal design.

A retrospective pre-post comparison of SPM metrics (ie, web and mobile log-ins, web-based applications and recertifications, and calls to update profile information) was conducted using monthly data collected in April 2018 and April 2019 (Table 3). The median age of the SNAP recipients was 25 to 44 years, and most were women (870,000/1,523,502, 57.11%). With regard to ethnic background, 27.83% (424,050/1,523,502) were Black only, 24.43% (372,245/1,523,502) were Hispanic and White only, 10.35% (157,723/1,523,502) were Black and Hispanic only, 11.23% (171,157/1,523,502) were multiethnic, 14.6% (222,495/1,523,502) were White, 10.29% (156,813/1,523,502) were Asian, and 1.25% (19,019/1,523,502) were of other ethnicities.

**Table 3:** Social Program Management universal access pre- and postimplementation system metrics.

Metric	Predeployment (April 2018)	Postdeployment (April 2019)	Percentage change (%)
System log-ins (web and mobile)	915,532	1,684,248	+83.96
Applications and recertifications received	33,421	40,198	+20.28
Profile update calls^a^	9696	17,547	+80.97

^a^Profile update calls to center staff are required to update profile information.

In the assessed periods, application rejections because of failure to provide documentation were reduced by 20% from 2674 in April 2018 to 2139 in April 2019 and center visits were reduced by 37% from 71,116 in April 2018 to 44,803 in April 2019. Of the 30,000 SNAP applications submitted in August 2019, 80% were submitted on the web through client-mobile devices via SPM’s universal access module. Client experience satisfaction was also measured with a web-based 5-star rating survey emailed to participants after they completed the application process. Responses (27,128) were collected with an average rating of 4.31 out of 5 (5 being the highest; 1 being the lowest) for the SNAP application and 4.44 out of 5 for the SNAP recertification.

### Case Report 2: Flexibility and Standardization in Digital Transformation

A US state’s health and human services department designed a program to improve the way state county departments provided services to families and allow caseworkers to spend less time on administrative tasks and more time helping individuals and families. Specifically, the state wanted real-time data sharing and aggregation across different health and human services divisions. At the time, most families were served through multiple categorical programs. Concurrently, the state wanted to limit the amount of system customization for each county yet be flexible enough to allow each to use legacy systems in a consolidated system managing all benefits and services until legacy systems could be sunset.

SPM was implemented in 2012, and information technology systems were modernized in more than 100 counties for 8 years. Deployments of SPM were designed to be interoperable with legacy systems so that incremental rollout could occur [15]. SPM has replaced or is in the process of replacing approximately 20 legacy systems with a single one. SPM’s common data model provides data sharing; therefore, caseworkers no longer need to enter data into multiple systems, spending less time on administrative tasks and more time assisting families. Participants’ administrative data can be viewed and shared in real time to support a holistic view of the client, their needs, and the analysis of progress toward goals and programmatic outcomes. Currently, at least 3.5 million individuals across the state have been provided benefits through SPM [16].

### Case Report 3: Data Aggregation, Sharing, and Collaboration

In a large city in Germany, 7 local districts and 40 regional agencies are responsible for protecting children from abuse. Two special government organization units support these districts and agencies in defining and monitoring policies and providing financial and technical resources. In an effort to provide better outcomes and serve clients more efficiently, the government sought to use SPM to improve processes for client intake, case management, and communication between agencies. Specific needs included improving upon reports of abuse, traditionally done through telephone and fax machines, and better data sharing and collaboration. The government leveraged the SPM platform to address these needs, including applications for verification of evidence and provider management for managing interactions between the government and local agencies, such as foster care. Implementation-specific modules included the social enterprise collaboration for supporting multidisciplinary teams and the child welfare module for managing child abuse cases. The solution was implemented in stages where standards for social services relevant to child abuse cases, such as foster care, were defined; an interface among SPM, legacy systems, and systems of local agencies was then deployed. In the second stage, modules for managing clients were implemented. These modules provided local agencies and the government with case management capabilities for documenting and sharing information related to interactions with children and families, services, contacts, worker visits, and judicial processes.

SPM allows caseworkers to view and manage a wide range of information in one place, from the initial receipt of an allegation through the final case outcome. This information enables bidirectional information sharing among local agencies and can then be used to document outcomes digitally. For example, when an allegation is made, supporting information such as police reports is automatically imported into SPM. As of June 2019, SPM in this city had 1300 users, 370,000 clients, and more than 200,000 cases and processed approximately 50,000 transactions per month since its implementation in 2014. This represents an average increase of 60% in the number of cases processed per year compared with the legacy system.

## Discussion

### Overview

We presented a configurable and modular system that delivers integrated cross-program and cross-agency solutions for needs-based and contribution-based social service programs [3,17-21]. Although social programs provide a wide variety of benefits across distinct and heterogeneous populations, fundamental needs are shared across programs that deliver services. SPM provides a set of functionalities that are generalizable and support government agencies and their beneficiaries across diverse program types and locations. These functionalities are configurable and adaptable to address the unique context of each social program.

The flexibility of SPM provides advantages in implementation and change management for the digital transformation of social programs. Most legacy social program systems have automated key program processes. Usually developed ad hoc, these older systems are complex, heterogeneous, and high maintenance. At the same time, these organizations tend to be risk-averse and often dependent on specific products or platforms [22]. Rather than imposing a need to redevelop the entire technology infrastructure, SPM can provide an interface between new solutions and legacy systems through modularity and open standards.

Finally, data aggregation and sharing are important for improving outcomes and tracking program success by reducing information gaps and providing a holistic view of clients. Previous research has demonstrated that integrated case management with a multidisciplinary approach may improve positive outcomes for clients [23]. SPM supports comprehensive data on service delivery for accountability and caseworker decision-making through a common data model. At the same time, a combination of business and infrastructure security mechanisms keeps client data protected and secure during program or agency collaboration, supporting trust in social programs.

This system description is limited in that the implementations described are on-premise solutions currently. Data quality and silos can limit the extent of insights and analytics, and how these data are presented influences decision-making. Improving program performance and achieving better health outcomes requires bringing together and presenting data visually to enhance decision-making abilities. These are common challenges for any enterprise-wide solution. We have made the platform more flexible and portable by moving more core processes of SPM to the cloud. We have released SPM to work on an open-source container application platform so that users can secure and use their data across multiple environments, including public and private clouds. We are also providing analytic capabilities across social programs and in a visual format at the point of decision-making to better assess the impact of social programs on health outcomes.

This viewpoint highlights the features of SPM with use cases selected to illustrate its generalizable features, such as benefits management, health and human services administration, and case coordination. These case studies were limited by organizations that were willing to share data and participate in the research. We were not able to design metric collection a priori, so the data for each use case are based on what could be provided by participating organizations.

### Conclusions

SPM is a user-centered, configurable, and flexible system designed to manage social program workflows. Its features and functionalities support the goals of social programs through improved service delivery to beneficiaries with functionalities and features for complex eligibility and entitlement, convenient access to services, complex case management, organizational and policy change management, and program transparency. More than 50 government organizations, 280,000 caseworkers, and 30 million beneficiaries are served through SPM, demonstrating the flexibility and scalability across social program types and settings in designing administrative systems that support a streamlined workflow.

## Appendix

Multimedia Appendix 1 Description of Social Program Management architecture.

## Abbreviations

SNAP: Supplemental Nutrition Assistance Program
SPM: Social Program Management
