# Supplementary material for: A System to Support Diverse Social Program Management
Source: JMIR Med Inform. 2021 Aug 30;9(8):e23219. doi: 10.2196/23219 (PMC8438610; doi:10.2196/23219)
Supplement: Multimedia Appendix 1 [file medinform_v9i8e23219_app1.docx]

**Supplement to Mckillop et al**

**“A System to Support Diverse Social Program Management”**

## Description of Social Program Management (SPM) Architecture

## Business Services

SPM provides support for efficient social program service delivery through common business services regardless of program type. These business services include: (i) a rules infrastructure with clear separation of the knowledge of what data are required by legislation and policy, and where that data are stored on the database, (ii) templates designed to ensure consistent, structured capture of information for caseworkers and for clients called Intelligent Evidence Gathering, (iii) a configurable rules-based decision matrix, called Decision Assist, designed to assure consistency and accuracy in rendering benefits decisions through visual display, (iv) an efficient search engine suitable for massive social program databases with functionality for searching incomplete information (since documentation may often be incomplete), (v) calendar functions to schedule and record notes for multi-disciplinary team meetings, and other program-related appointments, and (vi) correspondence management to enable organizations to manage and record various forms of communication (email, Pro forma, MS Word™, and communications issued outside SPM or received by the organization).

## Data Model

The data model is the flexible structural foundation for all SPM applications since it is built using stateless and atomic business objects which can be deployed as Web services. It is embodied as a metamodel containing over 500 logical entities and relationships centered around the client and household that support social program business processes for both needs-based and contributions-based programs for social services and workforce services. The data model is specified using the Unified Modeling Language for standardization.

## Technical Services

SPM business services and modules are supported by technical services which aim to standardize and support system maintainability, resulting in a reduced set of skills needed by system developers—an important feature in the resource-limited settings that social programs operate. These technical services include: (i) open-source standards and enterprise application integration connectors for external system interfaces, (ii) financial adapters for defining a generic interface between SPM and external financial systems, (iii) web services providing a standardized way of integrating web-based applications, (iv) security and authentication, both of which are important given the often sensitive nature of client data, (v) multi-channel system access (e.g., desktop or mobile), and (vi) application programming interfaces (APIs), acting as a technology abstraction layer.

## Administration application

To effectively leverage these business services in a highly dynamic environment, the administration suite supports routine system changes. Through a centralized web-based application, SPM provides the functionality required for configuring a wide range of organizational parameters to enable non-technical agency personnel to implement SPM and administration changes. Such changes may involve programs, rules, services, and activities provided by the organization, as well as the users, workgroups, and business processes managed within the system.
